# Evaluation of Thymic Changes after Median Sternotomy in Children

**Published:** 2014-05

**Authors:** Karmella Kamali, Mohammad Ghahartars, Ahmad Ali Amirghofran

**Affiliations:** Medical Imaging Research Center, Nemazee Hospital, Shiraz University of Medical Sciences, Shiraz, Iran

**Keywords:** Magnetic resonance imaging, Tetralogy of fallot, Median sternotomy, Thymus

## Abstract

In patients who undergo median sternotomy to treat congenital heart diseases, a thymectomy is performed to yield better access to the cardiac system. In this study we have used MRI to evaluate the changes in size, shape and location of the thymus after midsternatomy. This case-control study was performed during 2011-2012 in Shiraz, Iran. Eligible participants between 5-17 years of age were divided into case and control groups (n=13 per group). Each participant underwent a median sternotomy at least one year prior to study entry. Participants were initially examined by a cardiologist and then referred for MRI. A radiologist examined all MRI images. The thymus was observed in all control group patients and in only 7 (53.8%) patients in the case group. There was a significant relationship noted in terms of mean age in the group whose thymus was visible and the group in which the thymus was not visible. We have observed no significant difference in thymic visibility between these two groups based on the mean age at midsternatomy. In pediatric patients undergoing cardiac surgery the possibility of remaining or regenerated thymic tissues may be evaluated by MRI. The remaining portion of the thymus may have any shape, size or location. Therefore, it can be misinterpreted as a mass if a patient’s previous surgical history and age at the time of surgery are not taken into consideration.

## Introduction

The thymus, as a specialized central lymphoid organ, is responsible for the growth, differentiation, and development of lymphocytes, in particular T lymphocytes. Anatomically, this organ is situated at the anterior superior mediastinum, exactly behind the sternum and in front of large blood vessels.^1^

The most common congenital cyanotic heart disease in adults is the tetralogy of Fallot (TOF).^2^ Complete correction of TOF is performed under cardiovascular bypass surgery. Long-term evidence regarding the prognosis of such patients is not available; however, most affected children are treated or at least become asymptomatic throughout childhood or at the beginning of adulthood.^3^

Access to the heart and large vessels is difficult in infancy and the first months of life because of the size of the thymus. Therefore in children with congenital heart disease, a thymectomy is performed in order to facilitate cardiac surgery. This procedure leads to ectopic thymus tissue and ultimately thymus hyperplasia in the mediastinum.^4^^,^^5^

Magnetic resonance imaging (MRI) is a suitable method for evaluating a normal or hyperplasic thymus and related tumors. When compared with computed tomography, it is safer because patients are not exposed to a higher dose of radiation.^6^^,^^7^

In patients who undergo median sternotomy because of congenital heart disease, thymectomy is performed to enable better access to the cardiac system. However, the main question is whether the thymus is able to regenerate after surgery. Therefore, considering the increasing prevalence of congenital heart diseases worldwide (including Iran) and few existing studies in this regard, it is necessary to evaluate the changes in the thymus after surgery and during follow-up to take the necessary therapeutic approaches. 

We aimed to evaluate the changes in size, shape, and location of the thymus after midsternatomy using MRI.

## Materials and Methods

This case-control study was performed during 2011-2012 in the MRI Center of Shahid Faghihi Hospital, affiliated with Shiraz University of Medical Sciences, Shiraz, Iran. Participants were selected according to the simple sample selection method. Eligible individuals were divided into case and control groups. The control group (n=13) consisted of individuals with no history of chest surgery or known illness who referred for MRI for any other reasons. The case group (n=13) consisted of patients with TOF who were 5-17 years of age and had undergone median sternotomy only once (complete correction) at least one year prior to the study. The one-year period was considered necessary in order to bypass any transient thymic hyperplasia that might occur in the first few months after surgery. The sample size was calculated using the simple calculation method. 

We excluded patients with any accompanying pathology such as DiGeorge syndrome, those who used steroids, or those who had recent infections over the previous two weeks.

After obtaining written informed consent and approval from the Ethics Committee at Shiraz University, patients were interviewed to complete the related questionnaire. The other required data were collected through a review of patients' surgery reports. The included patients were subsequently examined by a cardiologist and referred for MRI. 

In order to study the effect of factors such as age and elapsed time from surgery we subdivided the participants into two groups - those in whom the thymus was visible through MRI and those in whom the thymus was not visible.

Imaging was done using a Siemens device (Siemens, Germany) with a magnetic field of 1.5 Tesla. The protocol for the obtained sequences performed by a single technician under the supervision of a radiology resident was as follows: axial HASTE sequence, axial T_2 _turbo, and axial in and out phase. We initially aimed to obtain sagittal in and out phase images. However, considering the longer imaging time and the lack of patients’ cooperation to control their respirations, we changed to the mentioned sequences.

All images were saved in the picture archiving and communication system (PACS) and evaluations were performed on a work station. All images and sequences were accurately examined by a single radiologist. The visibility, shape (round, smooth, lobulated, regular, and irregular borders), tissue heterogeneity and homogeneity, size (biggest size in the transverse, anterior-posterior directions and height), and place of the organ as well as ectopic or hyperplasic tissue were accurately examined. All images were carefully examined for any random findings. 

Data were analyzed using SPSS software, version 15. Mann-Whitney U and Fisher’s exact tests were used as appropriate. A P value<0.05 was considered statistically significant.

## Results

In the case group, there were 6 girls and 7 boys (median age: 7 years, range: 5-17 years). The control group consisted of 6 boys and 7 girls (median age: 12 years, range: 7-17 years). The patients’ ages ranged from 1-14 years at the time they underwent median sternotomy. The elapsed time after surgery varied from 2-7 years. 

The thymus was easily observed in all participants in the control group on axial HASTE images compared with only 7 (53.8%) patients in the case group (P=0.015). We found that gender did not have a significant effect on visualization of the thymus (P=0.695). The mean±SD time elapsed from surgery in those whose thymus was visible through imaging was 3.14±1.77 years and in those whose thymus was not visible, it was 3±0.894 years (P=0.73, Mann Whitney). For re-evaluation we divided the patients into two groups based on the time elapsed from surgery (2 years and over 2 years). There was no significant relationship between these two groups (P=1, Fisher’s exact test).

There was a significant relationship in terms of mean age between the group in which the thymus was visible (9.7±4.23 years) compared to the group in which the thymus was not visible (7±1.14 years, P=0.007). The age range of the latter group was 5-9 years (median: 7 years) compared with 5-17 years (median: 10 years) in the group in which the thymus was visible. There was no significant difference regarding the visibility of the thymus based on the mean age at which the patient underwent surgery in the non-visible thymus group (4±2 years) versus the group with a visible thymus (6.43±4.23 years).

None of the patients in the case group had a normal size thymus, nor was the size close to normal. 

## Discussion

In our study, the thymus was seen in all patients in the control group. In the only previous study in this regard, the thymus was seen in 92% of the patients in control group and in the remaining patients the thymus was not visible for unknown reasons.^8^ In our case group the thymus was visible in 53.8% of the patients by axial HASTE image. This finding suggested that more than half of the children had either a persistent or regenerated thymus after open cardiac surgery which could be attributed to the type of patient selection. We have selected patients with a higher age (over 5 years) or those who had underwent surgery only once by a single surgeon using a similar method. As the thymus consists of a wide variety of shapes and sizes, prediction of the shape and size of any remaining thymic tissues can be difficult. These three factors of a higher age over 5 years, only one surgery, and similar surgical method can enhance the visualization of the remaining portion of the thymus after mid-sternatomy, however in the superior mediastinum this leads to increased thymic identification in comparison with a report by MacDonald and Mackenzic who have reported 29% thymus identification.^8^

A unique feature of our study was that we chose only patients with TOF rather than different types of ongenital heart disease (CHD). Patients were operated on by a single surgeon using a similar method. All images were obtained by the same device with a standard protocol and by a single technician. Images were examined under the supervision of a radiology resident and the undesirable images were repeated until an acceptable image was obtained. 

Less than half of the children in our study, regardless of their age at sternotomy, did not have an identifiable thymus according to MRI after surgery. 

In our study the control group consisted of individuals with no history of chest surgery or known illness which referred to the MRI center for other reasons.

In most patients in the case group the thymus was clearly smaller. The signal was heterogeneous in 3 patients and the shape of the thymus was irregular in 5 patients. These were normal changes after surgery, thus the remainder of the thymus could be of any shape and dimension and located in any part of the mediastinum.

An important question which arises is why the thymus is seen in some patients after median sternotomy. The best explanation can be the use of different techniques during surgery. A study has reported a weak, although not statistically significant relation between the performance of cardiac surgery at a younger age and the presence of a visible thymus on subsequent imaging studies.^8^ At younger ages the gland’s tissue is situated mostly in the lateral sections and therefore less tissue is needed to be removed in order to reach the heart. However, we have found no relationship between age at which surgery is performed and subsequent visualization of the thymus. The method of thymus removal is not usually mentioned in patients’ records. This issue remains controversial and it is necessary to conduct additional related studies.

We found a significant relationship between patient’s age at the time of MRI and visibility of the thymus, which was not found in the previous study. The mean age of the patients in whom the thymus was visible was higher. This finding could be attributed to the age of the selected patients because in younger patients the surgeon should remove more thymic tissues compared to older patients. Considering the larger size of the thymus in adolescence, after surgery the thymic residue is larger and the peripheral part of the thymus could remain in place even after removal of the thymus compared with younger patients whose entire thymus gland is removed.

Several studies have shown that patients who undergo sternotomy and thymectomy experience long-term reduction in the amount of T lymphocytes and a disruption in their function.^9^^,^^10^ However, the long-term effects of reduction in the active tissue of the thymus and T lymphocytes on children who undergo related surgeries has yet to be evaluated. It is widely known that children who lack a thymus gland during the fetal period (DiGeorge syndrome) experience complete deficiency and dysfunction in T lymphocytes and have severe cellular immune deficiency. In patients who undergo sternotomy or thymectomy, it is possible that T lymphocytes may mature outside the thymus and the remaining tissue may suffice. However, the long-term effects of this defect under certain conditions such as infections have not been proven. Therefore, designing a long-term study to assess different groups of patients who have undergone median sternotomy can be beneficial.

A limitation of the current study was the inability to evaluate the T-cell counts for our patients. However none of the patients in the case group had any important health problems or developed malignant diseases. The long-term risk of reduced/absent thymic tissue in this population with respect to infectious or malignant diseases should be evaluated. We recommend that prospective studies be conducted.

## Conclusion

In pediatric patients who undergo cardiac surgery the possibility of remained or regenerated thymic tissues should be evaluated using MRI. The remaining portion of the thymus could have any shape, size, or location. Therefore, it could be misinterpreted as a mass if the history of previous surgeries and patient's age at the time of surgery has not been considered. 
